# Transient depression of myocardial function after influenza virus infection: A study of echocardiographic tissue imaging

**DOI:** 10.1371/journal.pone.0221628

**Published:** 2019-08-23

**Authors:** Takahide Ito, Kanako Akamatsu, Shu-ichi Fujita, Yumiko Kanzaki, Akira Ukimura, Masaaki Hoshiga

**Affiliations:** 1 Department of Cardiology, Osaka Medical College, Takatsuki, Osaka, Japan; 2 Department of General Internal Medicine, Osaka Medical College, Takatsuki, Osaka, Japan; Scuola Superiore Sant'Anna, ITALY

## Abstract

**Background:**

Influenza virus infection (IVI) was reported to be associated with minor cardiac changes, mostly those detected on electrocardiogram with and without elevated blood markers of myocardial injury; however, the characteristics of myocardial involvement in association with IVI are poorly understood. This study used echocardiographic tissue imaging (tissue Doppler, strain, and strain rate) to evaluate changes in left atrial (LA) and left ventricular (LV) myocardial function after IVI.

**Methods and results:**

We examined 20 adult individuals (mean age, 43 years) at 2 and 4 weeks after diagnosis of IVI. For myocardial functional variables, we obtained LV global longitudinal strain (GLS), LV early diastolic strain rate (e'sr), LA strain, and LA stiffness (E/e’/LA strain), in addition to data on tissue Doppler (s’, e’, and a’) and myocardial performance index. Blood markers of myocardial injury were also examined. During follow-up, there were no significant changes in global chamber function such as LV ejection fraction, E/e’, and LA volume. However, significant changes in myocardial function were observed, namely, in s’ (8.0 ± 1.6 cm/s to 9.3 ± 1.5 cm/s; p = 0.01), e’ (10.2 ± 2.8 cm/s to 11.4 ± 3.0 cm/s; p < 0.001), e’sr (1.43 ± 0.44 1/s to 1.59 ± 0.43 1/s; p = 0.005), and LA strain (35 ± 8% to 40 ± 12%; p = 0.025), and the myocardial performance index (0.52 ± 0.20 to 0.38 ± 0.09; p = 0.009), but not in a’, LA stiffness, or GLS. Cardiac troponin T and creatinine kinase isoenzyme MB were not elevated significantly at any examination.

**Conclusions:**

Myocardial dysfunction during IVI recovery appeared to be transient particularly in the absence of myocardial injury. Echocardiographic tissue imaging may be useful to detect subclinical cardiac changes in association with IVI.

## Introduction

Influenza virus infection (IVI) results in cardiac involvement up to 12% of cases [1]. The clinical profile of cardiac manifestations associated with IVI varies from transient electrocardiographic abnormalities to fulminant myocarditis requiring mechanical life-support [2,3]. Studies of patients during and after IVI noted ST-segment changes [2,4,5], elevations of the markers of myocardial injury, such as creatine kinase isoenzyme MB (CKMB), and impairment of global and regional left ventricular (LV) motions [6–8]. However, in IVI, abnormal findings obtained with these noninvasive modalities do not readily reveal the presence of substantial myocardial damage in the absence of concomitant elevation of cardiac troponin levels, and little information is available on the cardiac effect of IVI at the level of myocardium. The only relevant study showed that in IVI patients, myocardial velocity profiles derived from tissue Doppler imaging were altered, a finding of which clearly differed from those in controls although no data on myocardial injury were obtained [9].

Compared with conventional techniques on echocardiography including tissue Doppler imaging, speckle tracking echocardiography (STE) is more accurate for evaluating subclinical LV dysfunction [10–12]. STE was also reported to enable to assess left atrial (LA) function for detecting various subclinical conditions [13–15]. Few studies have used measures of echocardiographic tissue imaging, including STE-derived strain and strain rate, to assess the association of cardiac function with IVI. The present study, using these techniques, evaluated changes in LA and LV myocardial function associated with IVI.

## Materials and methods

### Study subjects

During the period from February 2014 through March 2018, we identified 114 adult individuals who had just recovered from IVI. Either type A or B IVI was diagnosed on the basis of a positive nasal swab specimen, as determined with a commercially available test kit, such as the QuickNavi-Ful kit (Denka Seiken, Tokyo, Japan). Among those individuals infected, 20 who completely underwent echocardiography and blood sampling at 2 and 4 weeks after IVI diagnosis were examined for the current study.

**Fig 1:**
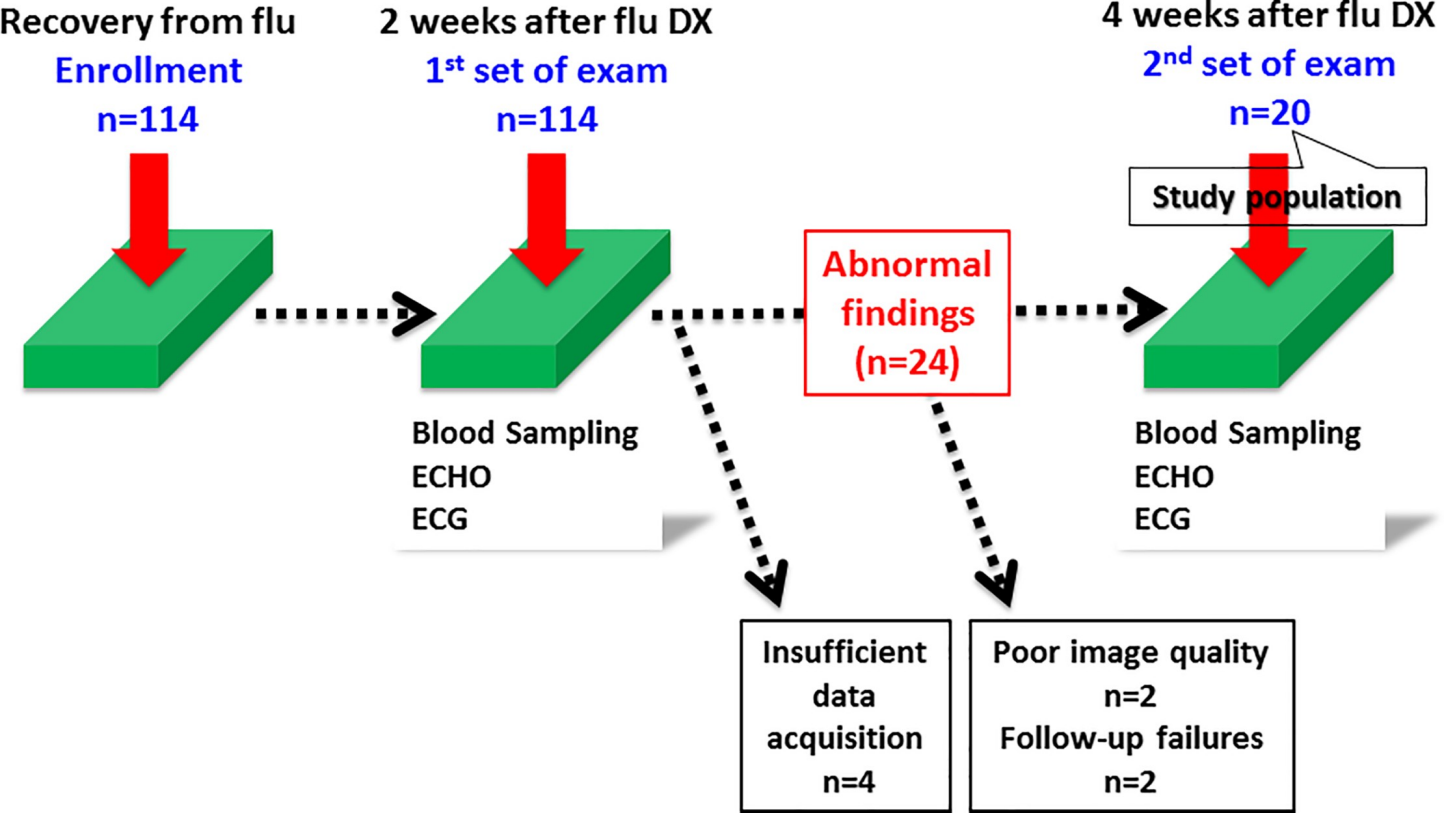
Study protocol and selection of the study subjects.

The present study was based on the data on our previous study [16], and thus was consistent with that study with respect to patient enrollment and study protocol. In the previous study, subjects recovering from IVI who presented 1 or more abnormal findings during the first assessment (electrocardiography for ST-T abnormalities, any arrhythmias, and others; echocardiography for reduced LV ejection fraction, diastolic dysfunction, and pericardial effusion; and elevated cardiac markers, namely, CKMB and/or cardiac troponin T) were advised to undergo a second set of assessment [16]. Clinical, echocardiographic, and biological characteristics at the initial set of examinations in patients who had any of the aforementioned abnormalities (n = 24) and in those who did not (n = 86) (4 excluded because of insufficient data acquisition) are presented in S1 Table, and how the 20 subjects were selected is shown in Fig 1.

This study was approved by the Institutional Ethics Committee of Osaka Medical College, and written informed consent was obtained from all participants.

### Blood sampling

Blood markers of cardiac injury, including CKMB, and high sensitive cardiac troponin T (cTnT) using HBsAgII quantII troponin T hs (Roche Diagnostics, Tokyo, Japan) were measured. Normal ranges for these markers in our laboratory are CKMB 7–15 U/L; and cTnT <0.014 ng/mL. Complete blood count and some contents of blood chemistry were also obtained.

### Standard echocardiography

Echocardiography was performed with commercially available ultrasound machines with the phased array probes (Vivid 7 Dimension or Vivid E9; GE Vingmed Ultrasound, Horten, Norway) at 2 and 4 weeks after IVI diagnosis. All measurements were performed by experienced sonographers blinded to background characteristics of the subjects. LA diameter, LV dimensions, and LV wall thickness were measured with M-mode method under 2-dimensional guidance. LV ejection fraction was calculated by the modified Simpson’s rule in apical 2- and 4-chamber views. LA volume was calculated with the disc method in apical 2- and 4-chamber views [17]. Pulsed Doppler was used to assess LV diastolic function; the early (E) and atrial filling (A) velocity, and their ratio (E/A), and deceleration time were obtained. Tissue Doppler-derived systolic (s’), early diastolic (e’), and atrial contraction (a’) velocities at the septal and lateral corners of the mitral annulus were measured and averaged. An averaged e’ and E/e’ were used as surrogates of LV relaxation and filling pressure, respectively [18]. Myocardial performance index (MPI) was also calculated with the pulsed Doppler sample volume placed at the LV outflow and inflow, as described previously [19]. Healthy adults have an MPI of <0.50 [20].

### STE

We used an EchoPAC workstation (GE Vingmed Ultrasound, Horten, Norway) to assess STE variables, including global longitudinal strain (GLS), global strain rate during early diastole (e’sr), and LA strain. The process to calculate STE-derived variables is followings. A couple of cardiac cycle loops for the apical 2-chamber, 3-chamber, and 4-chamber views (frame rate 80 to 100 frames per second) were stored in the workstation. At the end-systolic frame for each image, with the timing of aortic valve closure defined, the STE software algorithm automatically traced LV wall borders and tracked them throughout the cardiac cycle. Ultimately, we obtained longitudinal strain curves for 17 myocardial segments, and the strain (%) for each segment was presented on a bull’s eye display. GLS is an averaged strain value at end-systole for those segments. Because GLS is a negative value, we used its absolute value, |x|, to simplify interpretation; a GLS of <17.3% was considered to be abnormally low [21]. The e’sr was calculated by averaging the values for peak strain rate during early diastole for the 3 apical views [22,23]. To measure LA strain, with apical 2- and 4-chamber views, the STE software algorithm was applied to the LA wall [13–15]. LA stiffness was calculated as averaged E/e’ divided by LA strain (E/e’/LA strain) [13].

### Subclinical systolic and diastolic function variables

Table 1 lists subclinical myocardial functional variables measured in the current study. The LV variables were averaged s’, averaged e’, MPI, GLS, and e’sr, and LA variables were averaged a’, LA strain, and LA stiffness.

**Table 1:** Variables used to assess subclinical myocardial function.

Pulsed Doppler	Tissue Doppler	Strain by STE	Strain rate by STE
MPI (LV systolic and diastolic)	s’ (LV systolic)	s’ (LV systolic)	e’sr (LV diastolic)
	e’ (LV diastolic)	LA strain/ stiffness (LA reservoir)	
	a’ (LA contraction)		

### Statistical analysis

Data are presented as mean ± standard deviation for continuous variables, and as numbers or percentages for categorical variables. Changes in clinical and echocardiographic variables between the first and second assessments were analyzed with the paired t-test. Categorical variables were compared with the chi-square test. McNemar’s test was used to assess changes in categorical variables. All of the statistical calculations were performed with SPSS for Windows ver. 24.0 (IBM, Chicago, IL). A p value of <0.05 was considered to indicate statistical significance.

## Results

### Patient characteristics

The demographic characteristics of the 20 subjects are presented in Table 2. There were 5 men and 15 women with a mean age of 43 years and several subjects had been treated for hypertension or thyroid disease. All subjects had received a seasonal influenza vaccine, and 19 had taken anti-influenza antiviral medication after IVI diagnosis.

**Table 2:** Demographic characteristics of the study individuals.

Variable	
Age	43±13
Female gender, n (%)	15 (75)
Influenza vaccination, n (%)	20 (100)
Any disorders accompanied	
Hypertension, n (%)	2 (10)
Thyroid disease, n (%)	2 (10)
Type of virus infected	
A, n (%)	15 (75)
B, n (%)	5 (25)
Anti-influenza drug used	
Laninamivir, n (%)	14 (70)
Oseltamivir, n, (%)	4 (20)
Peramivir, n (%)	1 (5)
Non-use, n (%)	1 (5)

### Changes in echocardiographic variables

Table 3 shows the results of the conventional echocardiographic assessment at 2 and 4 weeks after diagnosis of IVI. During follow-up, no changes were found in global LA- or LV-related variables such as LV ejection fraction, E/A, and LA volume. For tissue Doppler variables, significant changes were observed in the averaged s’ (p = 0.01) and averaged e’ (p < 0.001), but not in E/e’. The MPI improved significantly (p = 0.009): the number of subjects with an MPI ≥0.50 decreased from 11 (55%) to 1 (5%) (p = 0.006).

**Table 3:** Conventional echocardiographic parameters at 2 and 4 weeks after diagnosis of IVI.

Variable	2 weeks	4 weeks	p
Heart rate (bpm)	65±13	67±9	0.27
LA diameter (mm)	34±4	34±4	1.0
LA volume (mL)	41±12	39±10	0.53
LA volume index (mL/m^2^)	25±7	24±6	0.57
LVEDD (mm)	46±3	47±3	0.32
LVESD (mm)	29±3	30±2	0.22
LVEF (%)	63±5	64±5	0.59
IVST (mm)	7±2	8±2	0.67
PWT (mm)	8±2	8±2	0.32
E (cm/s)	71±15	78±15	0.037
A (cm/s)	56±16	58±21	0.67
E/A	1.34±0.50	1.50±0.54	0.15
Deceleration time (ms)	190±39	200±41	0.22
Lateral s’ (cm/s)	9.1±2.4	10.5±2.3	0.04
Septal s’ (cm/s)	6.9±1.1	8.0±1.0	0.002
Avaraged s’ (cm/s)	8.0±1.6	9.3±1.5	0.01
Lateral e’ (cm/s)	11.5±3.3	12.9±3.6	0.009
Septal e’ (cm/s)	8.9±2.8	9.9±2.5	0.018
Averaged e’ (cm/s)	10.2±2.8	11.4±3.0	<0.001
Lateral a’ (cm/s)	8.7±2.1	8.9±2.8	0.69
Septal a’ (cm/s)	7.7±2.0	8.5±2.1	0.078
Averaged a’ (cm/s)	8.2±1.7	8.7±2.0	0.23
Averaged E/e’	7.4±2.4	7.1±1.9	0.59
MPI	0.52±0.19	0.39±0.09	0.009

Variables were expressed as mean ± standard deviation. IVST = Interventricular septum thickness, LVEF = LV ejection fraction, LVEDD = LV end-diastolic dimension, LVESD = LV end-systolic dimension, MPI = myocardial performance index, PWT = posterior wall thickness.

**Fig 2:**
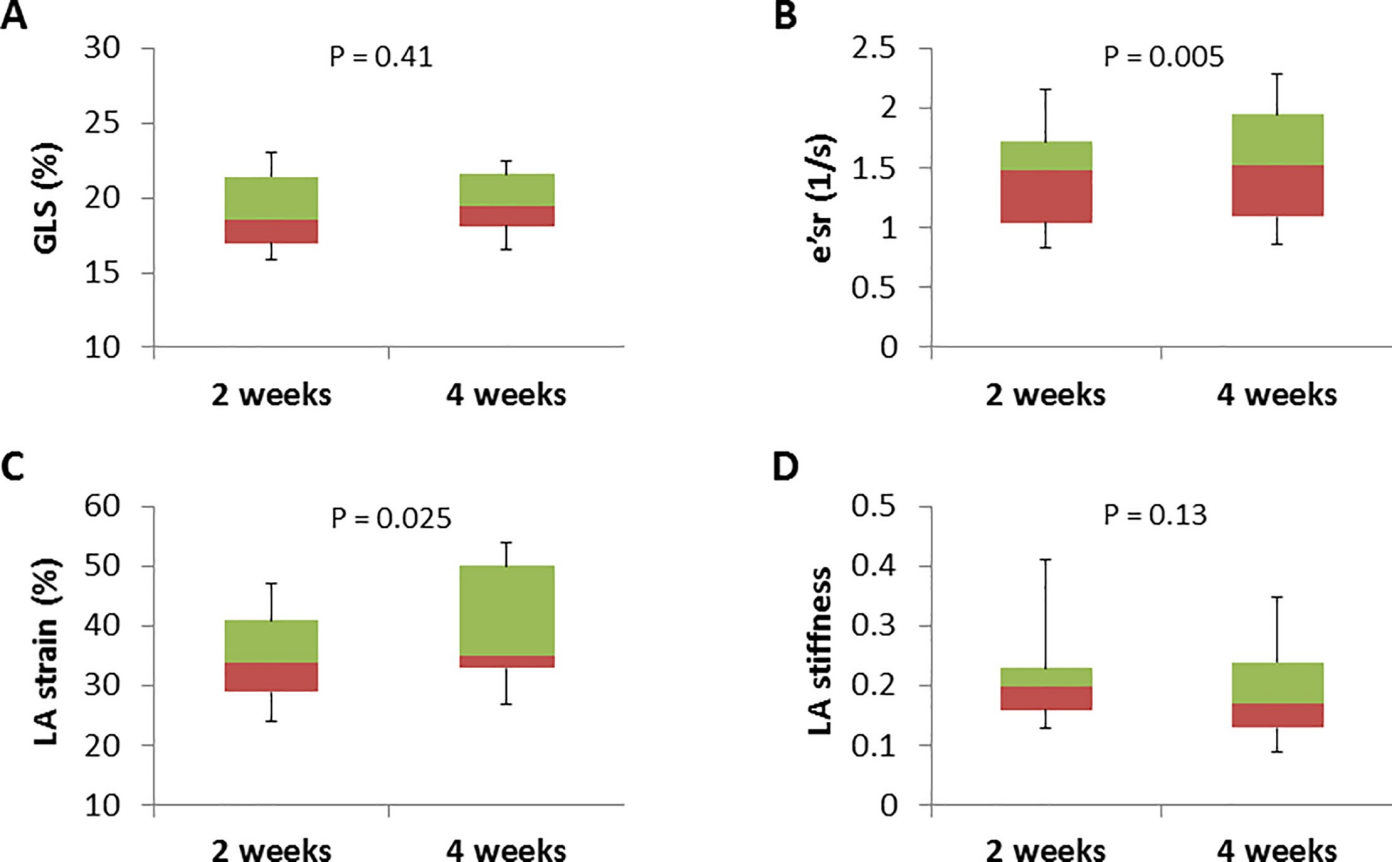
Comparisons of the STE-derived variables at 2 and 4 weeks after diagnosis of IVI.

Fig 2 compares the STE-derived variables at 2 and 4 weeks after IVI diagnosis. GLS did not change (19.2 ± 2.6% to 19.6 ± 2.3%; p = 0.41), and the number of subjects with an abnormal GLS (<17.3%) decreased from 6 (30%) to 2 (10%) (p = 0.22). In contrast, significant increases were found in e’sr (1.43 ± 0.44 1/s to 1.59 ± 0.43 1/s; p = 0.005) and LA strain (35 ± 8% to 40 ± 12%; p = 0.025). The LA stiffness decreased during follow-up, but the change was not statistically significant (0.22 ± 0.10 to 0.20 ± 0.10; p = 0.13).

**Fig 3:**
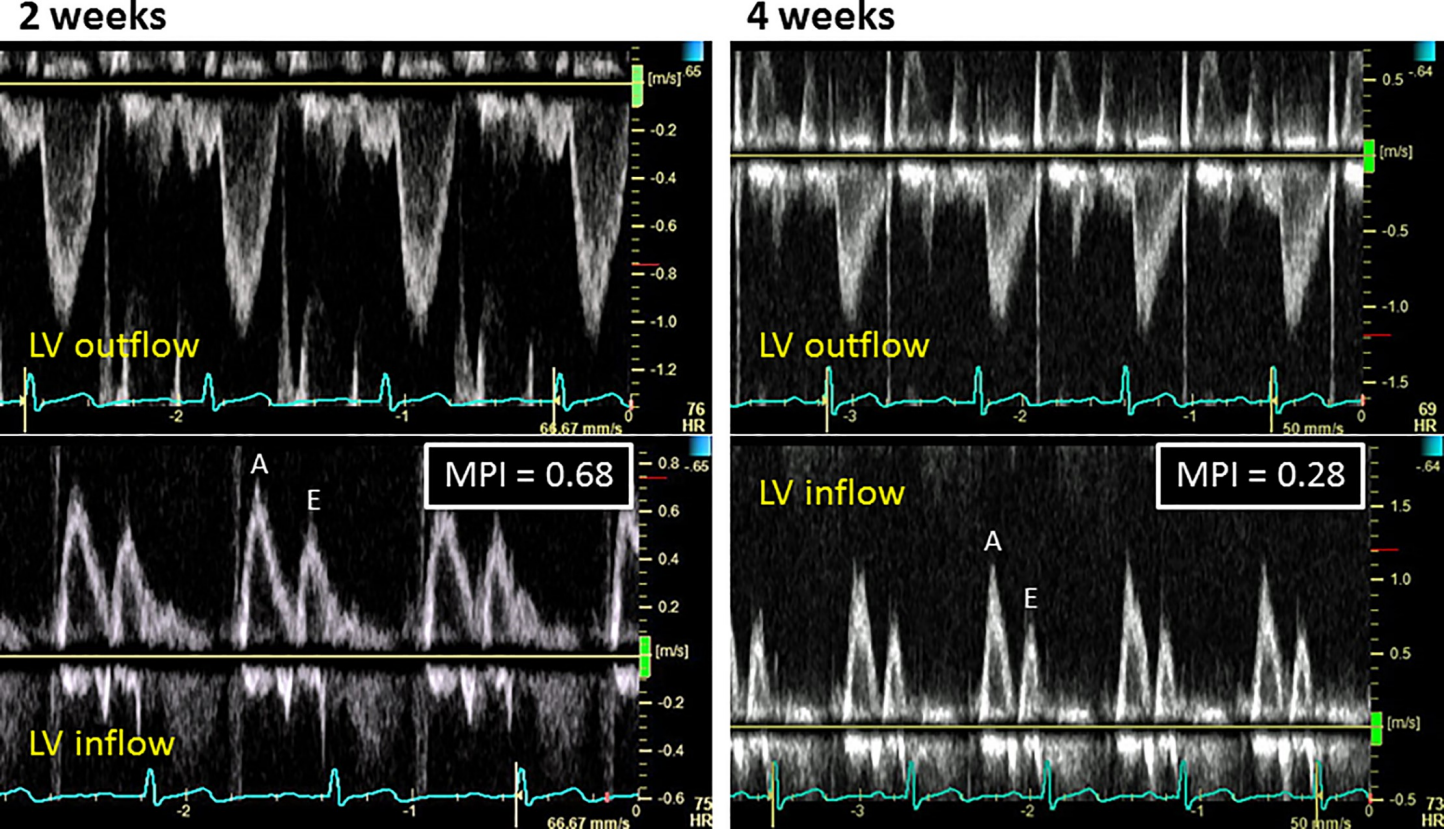
Doppler LV outflow (top) and inflow (bottom) profiles used to measure MPI at 2 and 4 weeks after diagnosis of IVI in a woman aged 42 years.

**Fig 4:**
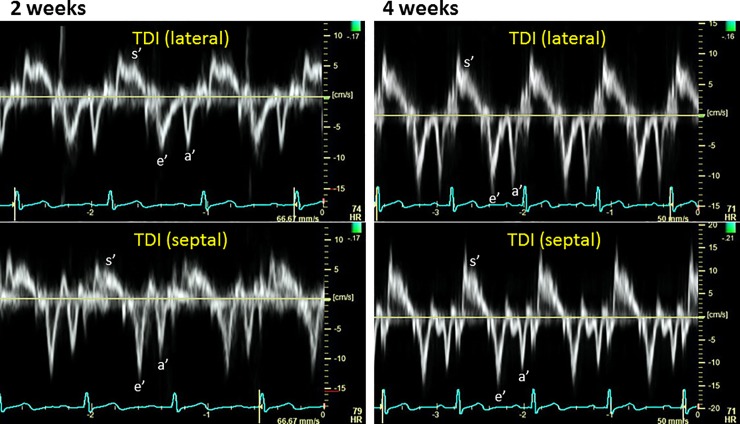
Tissue Doppler profiles for the lateral (top) and septal (bottom) corners of the mitral annulus, for the patient as in Fig 3.

**Fig 5:**
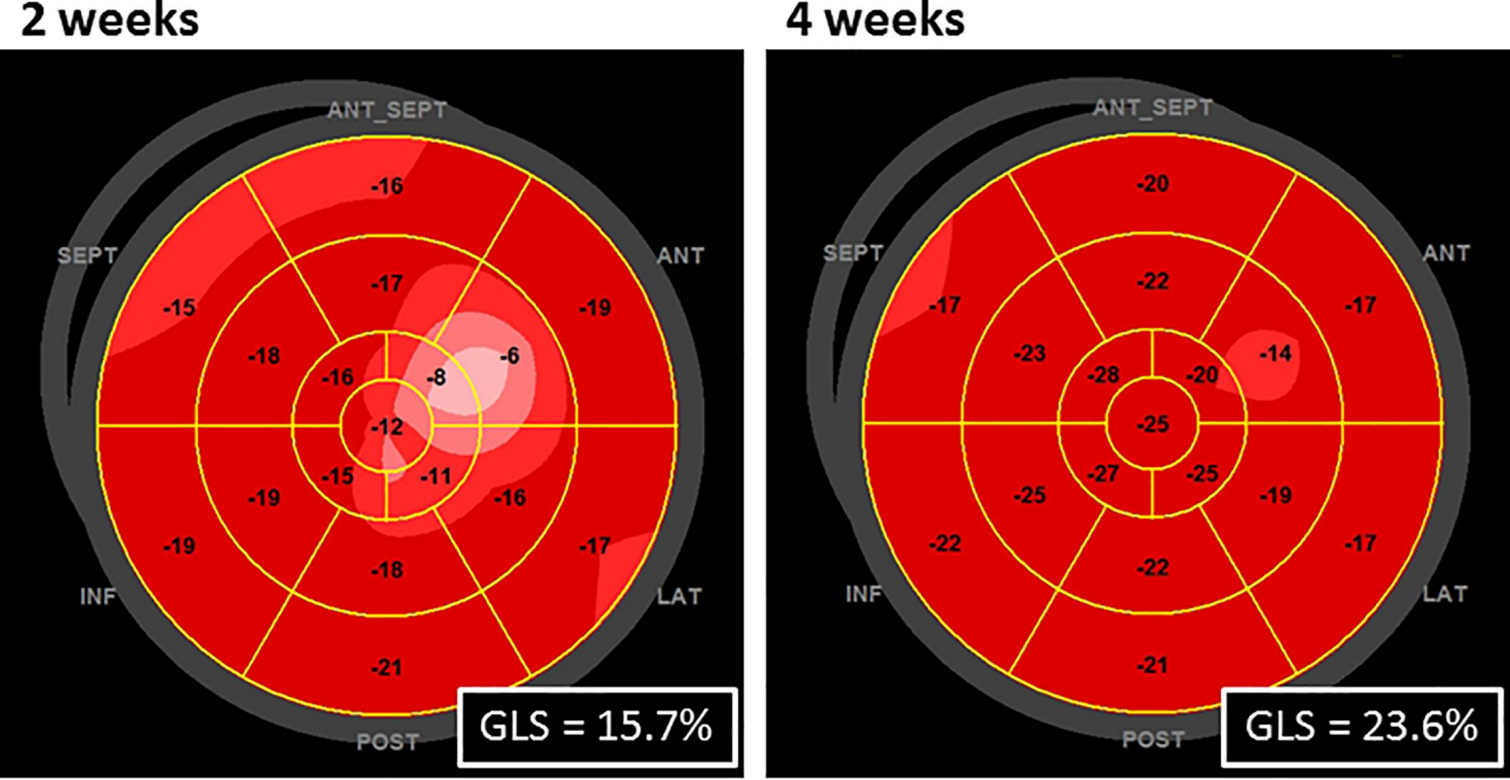
Bull’s eye GLS display for each LV segment for the patient as in Fig 3.

**Fig 6:**
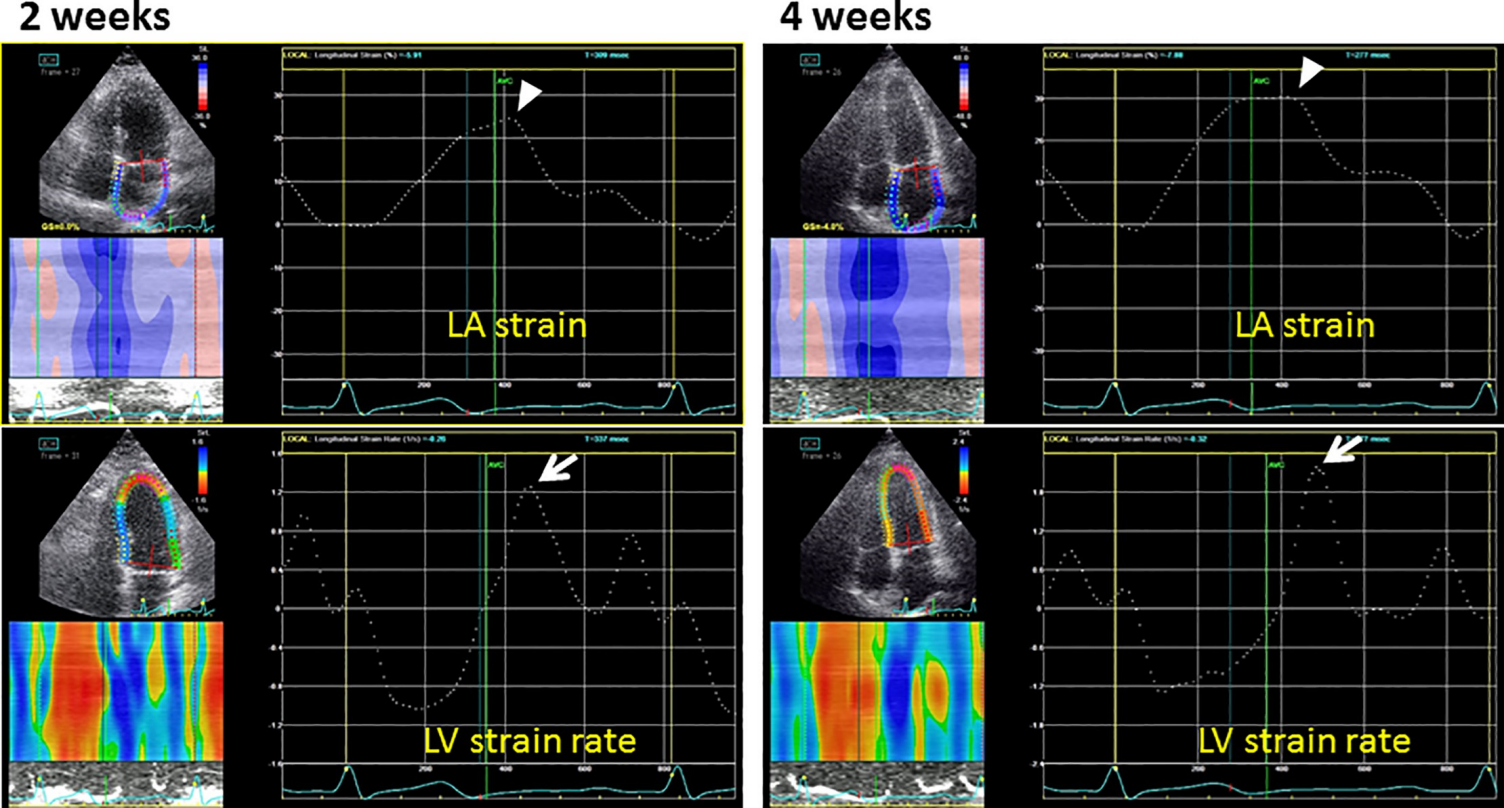
Strain and strain rate curves for the LA (top) and LV (bottom) to assess LA strain (arrows) and e’sr (arrow heads), respectively, for the patient as in Fig 3.

Figs 3–6 show representative recordings of echocardiographic tissue imaging.

### Changes in the markers of myocardial injury

Table 4 shows changes in the blood markers of myocardial injury and other blood sampling results including complete blood count and blood chemistry. At 2 weeks after IVI diagnosis, 3 subjects had CKMB higher than 15 U/L, but the elevation did not seem to be clinically significant. The cTnT was not elevated in any subjects at either assessment. On the other hand, no significant changes were observed in inflammatory markers such as white blood cell count and C-reactive protein, or in B-type natriuretic peptide. Only the platelet count was significantly reduced during follow-up.

**Table 4:** Biological makers from blood sampling obtained at 2 and 4 weeks after diagnosis of IVI.

Variable	2 weeks	4 weeks	p
Markers of cardiac injury			
CKMB (U/L)	10±7	9±6	0.16
Range of CKMB (U/L)	4–36	4–30	-
cTnT ≥0.014 ng/mL, n (%)	0 (0%)	0 (0%)	-
Range of cTnT (ng/mL)	<0.003–0.008	<0.003–0.007	-
Complete blood count			
White blood cell (/μL)	6186±1611	6003±1553	0.35
Red blood cell (10^6^/μL)	4.46±0.49	4.44±0.42	0.17
Hemoglobin (g/dL)	13.5±1.4	13.3±1.3	0.20
Neutrophil (%)	59.7±9.3	59.0±9.3	0.61
Eosinophil (%)	2.2±1.8	2.1±1.4	0.80
Lymphocyte (%)	30.5±8.4	31.2±8.0	0.55
Platelet (10^3^/μL)	269±65	235±50	<0.001
Blood chemistry			
AST (U/L)	19±6	20±5	0.66
ALT (U/L)	20±11	19±10	0.84
CK (U/L)	89±44	104±87	0.24
Range of CK (U/L)	30–238	47–483	-
BUN (mg/dL)	12±5	13±3	0.58
Creatinine (mg/mL)	0.71±0.18	0.74±0.28	0.55
BNP >18.4 pg/mL, n (%)	6 (30)	8 (40)	0.25
Range of BNP (pg/mL)	<5.8–51.2	<5.8–28.2	-
CRP >0.25 mg/dL, n (%)	3 (15)	2 (10)	1.0
Range of CRP (mg/dL)	<0.01–0.79	<0.01–1.02	-

Continuous variables were expressed as mean ± standard deviation and categorical variables as percentages. ALT = alanine aminotransferase, AST = aspartate aminotransferase, BNP = B-type natriuretic peptide, BUN = blood urea nitrogen, CK = creatine kinase, CKMB = creatine kinase isoenzyme MB, CRP = C-reactive protein, cTnT = cardiac troponin T.

## Discussion

### Main findings

The present study found that myocardial function was depressed during recovery from IVI, which was not associated with elevations of the markers of myocardial injury. This finding suggests that cardiac involvement during IVI is transient as long as usual healing course of IVI is taken without any further deterioration of the clinical pictures. On the other hand, our findings indicate that echocardiographic tissue imaging enable to detect subtle and reversible myocardial changes in association with IVI.

### Previous studies using echocardiography in IVI

Few studies have used echocardiography to examine IVI-related cardiac abnormalities. Ison et al examined 30 asymptomatic IVI patients with echocardiography at 2, 11 and 28 days after presentation and found no global or regional wall motion abnormalities in any patients at any point, despite some patients exhibiting slight elevations of CKMB [4]. Among 41 patients with serologically confirmed IVI, Karjalainen et al found that 9 had abnormal electrocardiographic findings and/or LV wall motion disturbance [6]; however, pre-existing wall motion abnormalities seemed to be implicated as a cause of IVI-related myocardial involvement.

MPI is an index of cardiac function that represents both contraction and relaxation of cardiac chambers [19] and is used not only to assess severity of heart diseases but also to detect subclinical dysfunction before development of overt heart disease [24–26]. In a study of 28 young patients hospitalized with IVI, Erden et al found that MPI and tissue Doppler s’ and e’ could differentiate IVI patients from age-matched controls without alterations in global systolic or diastolic function [9]. Their findings were consistent with ours in that the percentage of patients with an MPI ≥0.5 (50% vs 56%) was similar.

Little is known about the role of STE in the evaluation of IVI-related myocarditis. Han et al evaluated cardiac injury caused by avian-origin influenza A virus (H7N9) infection and observed that patients who developed cardiovascular complications, including overt heart failure, had elevated cardiac troponin I and decreased GLS [27]. With the results of their study taken into account, our finding of GLS remaining unchanged during follow-up may not be surprising.

Another STE-derived variable we used here, e’sr, may be better than tissue Doppler e’ for assessing abnormalities of LV relaxation [22], because e’sr covers the entire endocardial motion throughout diastole, during which the LV chamber not only expands but also untwists [22, 23]. Our observation that e’sr depression was greater at 2 weeks than at 4 weeks after IVI diagnosis implicates the presence of transient, but subtle, impairment of myocardial function, further supporting the findings of changes in s’, e’, and MPI.

In the present study, some of the myocardial variables (those derived from tissue Doppler and strain rate) were shown to reach statistical significance but others (GLS and LA strain) did not. This may mostly be related to the small number of subjects. However, we believe that tissue Doppler and strain rate are more sensitive in detecting subclinical disease because they, from a methodological viewpoint, represent dynamic, instantaneous function, whereas GLS represents a stationary condition similar to LV ejection fraction.

### Presumed mechanism of IVI-related myocardial dysfunction

The cTnT has been found to be a more sensitive maker for detecting biopsy-proven myocarditis than conventional markers including CKMB [28]. In this regard, no myocardial damage was considered to be present in any of our subjects, although some showed abnormal findings on echocardiographic tissue imaging. We cannot explain this discrepancy since no data are available that would allow direct comparison of cardiac troponin and echocardiographic measurements for diagnosing myocarditis. Myocardial dysfunction associated with IVI may be related to transient impairment of intracellular calcium handling that results from lingering exposure to various inflammatory cytokines [9] or to transient hypoxia-induced mitochondrial dysfunction [29].

With sepsis, wherein a variety of inflammatory cytokines go around within the human body, there have been several reports on myocardial dysfunction with otherwise normal chamber function as assessed by echocardiographic tissue imaging [30–32]. Among 47 patients hospitalized with severe sepsis or septic shock, 17 were shown to have worsening of MPI during the first 24 hours of hospitalization, which was associated with elevated in-hospital mortality [30]. Likewise, in septic patients, reduced GLS was shown to be associated with higher mortality rate [31]. Mechanisms underlying altered MPI and GLS in the setting of sepsis remained to clearly be elucidated, presumably due to the heterogeneity of study population. From the experimental viewpoint, however, there have been several mechanisms proposed including excessive formation of nitric oxide (NO), reactive oxygen species (ROS), or the exposure of myocardial cells to inflammatory cytokines such as tumor necrosis factor-alpha (TNF-α) [32].

Nevertheless, most of our subjects were asymptomatic and did not seem to have significant hemodynamic or inflammatory findings. Thus, the aforementioned mechanisms were unlikely to exert on the myocardial function, although there was a possibility that certain myocardial depressant factors circulating had an influence on cardiac function far beyond the acute phase. Also, reduction of platelet count during follow-up (Table 4) might contribute in some way to the changes in myocardial function. A recent study using tissue Doppler has reported that in patients with coronary artery disease, platelet activation can be involved with impairment of diastolic function [33]. A large volume of participants would be required to address this relationship.

### Limitations

This study has limitations inherent to a single-center, observational study with the small sample size. Particularly, the study population was too small to represent the entire spectrum of cardiac dysfunction and patterns of recovery associated with IVI. Also, it was hard to explain the clinical implications of our study and how information obtained could be used in clinical practice. We did not obtain the data set at the time of (or before) IVI diagnosis (Fig 1). Only the earliest evaluation at 2 weeks from IVI diagnosis might miss not only assessing for cardiac abnormalities occurring in the very early phases of the disease, but also comparing with pre-infection values to ascertain whether any abnormality was pre-existing or due to the infection.

Other biomarkers for detecting myocardial injury such as cardiac troponin I and myosin light chain I were not measured in this study. However, most of the previous studies of IVI-related myocarditis found no positive response to troponin I or T [4,8], and myosin light chain I elevation appeared to occur in relatively older patients [7]. Immunobiological analysis against a myocardial specimen is the current diagnostic gold standard for defining myocarditis [34], and a positive result is not always accompanied by elevated cardiac troponin levels. However, invasive procedures are impractical for patients with common, easily diagnosed, and usually self-limiting disorders. Finally, we did not perform blood pressure measurement and its influence on hemodynamic parameters is unknown.

Despite those limitations, our results may indicate that especially in terms of the need of biopsy to diagnose myocarditis, echocardiographic tissue imaging becomes a noninvasive diagnostic tool for identifying myocardial dysfunction in IVI patients.

## Conclusions

We used echocardiographic tissue imaging to evaluate myocardial function in adult individuals with IVI. During early recovery from IVI, myocardial function was depressed without alteration of global chamber function. Given that no evidence of myocardial injury was observed, this finding appeared to exhibit subtle myocardial changes that are reversible. Further studies, recruiting a larger number of participants, are necessary to confirm our results and identify thresholds at which any abnormal values on echocardiographic tissue imaging alter the clinical management in IVI patients.

## Supporting information

S1 Table Clinical, echocardiographic, and biological characteristics at the initial set of examinations in patients who had any abnormalities on ECG, echo, or blood sampling (n = 24) vs. those who did not (n = 86). (DOCX)

S2 Table Raw data of patients who underwent follow-up examinations (n = 20) in addition to those of patients who had abnormal findings at the initial examinations but could not receive follow-up examinations (n = 4). (XLSX)
