# Supplementary material for: Transient depression of myocardial function after influenza virus infection: A study of echocardiographic tissue imaging
Source: PLoS One. 2019 Aug 23;14(8):e0221628. doi: 10.1371/journal.pone.0221628 (PMC6707632; doi:10.1371/journal.pone.0221628)
Supplement: S1 Table — (DOCX) [file pone.0221628.s001.docx]

Clinical, echocardiographic, and biological characteristics at the initial set of examinations in patients who had any abnormalities on ECG, echo, or blood sampling (n=24) vs. those who did not (n=86).

| Variable | Cardiac findings absent (n=86) | Cardiac findings present (n=24) | p |
| --- | --- | --- | --- |
| Age | 35±10 | 43±12 | 0.012* |
| Female gender, n (%) | 64 (74) | 16 (67) | 0.46 |
| Influenza vaccination, n (%) | 86 (100) | 23 (96) | 0.079 |
| Any disorders accompanied |  |  |  |
| Hypertension, n (%) | 1 (0) | 2 (8) |  |
| Thyroid disease, n (%) | 0 (0) | 2 (8) |  |
| Diabetes mellitus, n (%) | 0 (0) | 1 (4) | <0.001 |
| Type of virus infected |  |  |  |
| A, n (%) | 77 (90) | 19 (79) |  |
| B, n (%) | 9 (10) | 5 (21) | 0.20 |
| Anti-influenza drug used |  |  |  |
| Laninamivir, n (%) | 52 (60) | 17 (71) |  |
| Oseltamivir, n (%) | 25 (29) | 5 (21) |  |
| Peramivir, n (%) | 3 (3) | 1 (4) |  |
| Zanamivir, n (%) | 4 (5) | 0 (0) |  |
| Non-use, n (%) | 2 (2) | 1 (4) | 0.56 |
| Echocardiographic variable | | | |
| Heart rate at echo (bpm) | 67±11 | 66±10 | 0.70 |
| LA diameter (mm) | 32±4 | 35±4 | 0.024* |
| LA volume (mL) | 35±13 | 39±12 | 0.21 |
| LA volume index (mL/m^2^) | 23±8 | 24±7 | 0.38 |
| LVEDD (mm) | 45±4 | 47±3 | 0.11 |
| LVESD (mm) | 29±3 | 30±3 | 0.092 |
| LVEF (%) | 65±5 | 63±5 | 0.081 |
| IVST (mm) | 7±1 | 7±2 | 0.38 |
| PWT (mm) | 7±1 | 8±1 | 0.022* |
| E (cm/s) | 85±17 | 72±18 | 0.003* |
| A (cm/s) | 49±13 | 54±17 | 0.10 |
| E/A | 1.85±0.62 | 1.46±0.61 | 0.005* |
| Deceleration time (ms) | 182±35 | 196±38 | 0.078 |
| Lateral s’ (cm/s) | 10.8±2.4 | 9.6±2.4 | 0.047* |
| Septal s’ (cm/s) | 8.6±1.3 | 7.4±1.6 | <0.001* |
| Averaged s’ (cm/s) | 9.7±1.6 | 8.5±1.8 | 0.003* |
| Lateral e’ (cm/s) | 15.6±3.3 | 12.7±4.2 | 0.004* |
| Septal e’ (cm/s) | 12.5±2.5 | 9.6±3.1 | <0.001* |
| Averaged e’ (cm/s) | 14.0±2.6 | 11.1±3.4 | <0.001* |
| Lateral a’ (cm/s) | 7.9±1.9 | 8.3±2.1 | 0.40 |
| Septal a’ (cm/s) | 7.9±1.8 | 7.8±2.0 | 0.96 |
| Averaged a’ (cm/s) | 7.9±1.6 | 8.1±1.7 | 0.63 |
| Averaged E/e’ | 6.1±1.2 | 6.9±2.3 | 0.024 |
| MPI | 0.34±0.12 | 0.49±0.20 | <0.001* |
| GLS (%) | 20.6±2.6 (n=80) | 19.3±2.5 (n=22) | 0.057* |
| LVEF <50%, n (%) | 0 (0) | 0 (0) |  |
| Pericardial effusion | 0 (0) | 2 (8) | 0.013 |
| Diastolic dysfunction, n (%) | 0 (0) | 14 (58) | <0.001 |
| ECG findings | | | |
| Normal, n (%) | 86 (100) | 15 (63) |  |
| ST-segment elevation | 0 (0) | 3 (13) |  |
| PAC | 0 (0) | 2 (8) |  |
| T-wave flattering | 0 (0) | 1 (4) |  |
| PVC | 0 (0) | 1 (4) |  |
| Right-bundle branch block | 0 (0) | 1 (4) |  |
| Poor R wave progression | 0 (0) | 1 (4) |  |
| High voltage | 0 (0) | 2 (8) |  |
| ST-segment depression | 0 (0) | 1 (4) |  |
| Markers for cardiac injury | | | |
| CKMB >15 U/L, n (%) | 0 (0) | 5 (21) | <0.001 |
| Range of CKMB (U/L) | 2 – 15 | 4 - 36 |  |
| cTnT >0.014 ng/mL, n (%) | 0 (0) | 0 (0) |  |
| Range of cTnT (ng/mL) | <0.003 – 0.006 | <0.003 – 0.010 |  |
| Complete blood test | | | |
| White blood cell (/μL) | 6037±1416 | 5997±1518 | 0.90 |
| Red blood cell (10^6^/μL) | 4.39±0.41 | 4.45±0.46 | 0.59 |
| Hemoglobin (g/dL) | 13.0±1.2 | 13.5±1.4 | 0.074 |
| Neutrophil (%) | 60.1±9.0 | 60.0±9.0 | 0.96 |
| Eosinophil (%) | 2.2±1.5 | 2.1±1.6 | 0.89 |
| Lymphocyte (%) | 30.7±7.4 | 30.4±8.0 | 0.89 |
| Platelet (10^3^/μL) | 296±67 | 273±61 | 0.14 |
| Blood chemistry | | | |
| AST (U/L) | 19±7 | 20±6 | 0.64 |
| ALT (U/L) | 18±11 | 19±11 | 0.64 |
| CK (U/L) | 89±44 | 104±87 | 0.24 |
| CK >153 U/L, n (%) | 5 (6) | 0 (0) | 0.12 |
| Range of CK (U/L) | 30 - 238 | 47 - 483 |  |
| BUN (mg/dL) | 12.4±3.4 | 12.4±4.5 | 0.97 |
| Creatinine (mg/mL) | 0.68±0.13 | 0.71±0.17 | 0.41 |
| BNP >18.4 pg/mL, n (%) | 8 (9) | 5 (21) | 0.14 |
| Range of BNP (pg/mL) | <5.8 – 51.2 | <5.8 – 28.2 |  |
| CRP > 0.25 mg/dL, n (%) | 4 (5) | 3 (13) | 0.20 |
| Range of CRP (mg/mL) | <0.01 – 0.79 | <0.01 – 1.02 | 0.20 |

Continuous variables were expressed as mean ± standard deviation and categorical variables as percentages. Comparisons of categorical variables were performed using the chi-square test or Fisher’s exact test whereas those of continuous variables in the groups were performed using unpaired two test or Kruskal Wallis test according to whether normally distributed or not, as tested by Welch test. 0. XX* indicates a comparison of variables that distributed non-normally each other.

ALT = alanine aminotransferase, AST = aspartate aminotransferase, BNP = B-type natriuretic peptide, BUN = blood urea nitrogen, CK = creatine kinase, CKMB = creatine kinase isoenzyme MB, CRP = C-reactive protein, cTnT = cardiac troponin T, GLS = global longitudinal strain, IVST = Interventricular septum thickness, LVEF = LV ejection fraction, LVEDD = LV end-diastolic dimension, LVESD = LV end-systolic dimension, MPI = myocardial performance index, PWT = posterior wall thickness.
